# Organoid Models for Precision Cancer Immunotherapy

**DOI:** 10.3389/fimmu.2022.770465

**Published:** 2022-04-05

**Authors:** Cai-Ping Sun, Huan-Rong Lan, Xing-Liang Fang, Xiao-Yun Yang, Ke-Tao Jin

**Affiliations:** ^1^ Department of Medical Oncology, Shaoxing People’s Hospital (Shaoxing Hospital, Zhejiang University School of Medicine), Shaoxing, China; ^2^ Department of Breast and Thyroid Surgery, Affiliated Jinhua Hospital, Zhejiang University School of Medicine, Jinhua, China; ^3^ Department of Hepatobiliary Surgery, Affiliated Hospital of Shaoxing University College of Medicine (Shaoxing Municipal Hospital), Shaoxing, China; ^4^ Department of Gastroenterology, Affiliated Jinhua Hospital, Zhejiang University School of Medicine, Jinhua, China; ^5^ Department of Colorectal Surgery, Affiliated Jinhua Hospital, Zhejiang University School of Medicine, Jinhua, China

**Keywords:** cancer, immunotherapy, organoid, precision medicine, tumor microenvironment

## Abstract

Cancer immunotherapy is exploited for the treatment of disease by modulating the immune system. Since the conventional *in vivo* animal and 2D *in vitro* models insufficiently recapitulate the complex tumor immune microenvironment (TIME) of the original tumor. In addition, due to the involvement of the immune system in cancer immunotherapy, more physiomimetic cancer models, such as patient-derived organoids (PDOs), are required to evaluate the efficacy of immunotherapy agents. On the other hand, the dynamic interactions between the neoplastic cells and non-neoplastic host components in the TIME can promote carcinogenesis, tumor metastasis, cancer progression, and drug resistance of cancer cells. Indeed, tumor organoid models can properly recapitulate the TIME by preserving endogenous stromal components including various immune cells, or by adding exogenous immune cells, cancer-associated fibroblasts (CAFs), vasculature, and other components. Therefore, organoid culture platforms could model immunotherapy responses and facilitate the immunotherapy preclinical testing. Here, we discuss the various organoid culture approaches for the modeling of TIME and the applications of complex tumor organoids in testing cancer immunotherapeutics and personalized cancer immunotherapy.

## Introduction

Immunotherapy is a type of cancer therapy that boosts the body’s immune system to fight cancer. The immune system uses a variety of mechanisms to identify and eradicate tumor cells, but many of them are inactivated in the tumor microenvironment (TME) during tumor development (1). Immunotherapy can be exploited to help the immune system for detection of neoplastic cells and triggering of the immune response, or promote an existing one against the tumor cells. Indeed, immunotherapies systemically boost the immune surveillance and/or locally regulate the tumor immune microenvironment (TIME) (2). These immunotherapies include oncolytic viruses, pattern recognition receptor (PRR)-targeted therapies, vaccines, tumor antigen-targeted monoclonal antibodies, adjuvants such as cytokines, or other cell signaling molecules (3–5). Immunotherapy approaches that have revolutionized conventional malignancy treatment include: 1) adoptive T cell therapies (ACT), such as T cell receptor (TCR)- and chimeric antigen receptor (CAR)-T cells, also bulk tumor-infiltrating lymphocyte (TIL) therapy (6–8) and 2) immune checkpoint inhibitors (ICIs) (9–11) including anti-CTLA-4 monoclonal antibodies and anti-PD-1/PD-L1 monoclonal antibodies that boost CD8+ T cell effector functions. Since the immune system is involved in cancer immunotherapy, therefor, efficient cancer models are required to test the effect of immunotherapy agents in a context where there are immune cells and other TME components. In addition, the dynamic interactions between the neoplastic cells and non-neoplastic host components in the TME can affect carcinogenesis, tumor metastasis, cancer progression, and drug resistance of cancer cells. Hence, conventional *in vivo* animal and 2D *in vitro* models are not suitable for immunotherapy because these models insufficiently recapitulate the complex tumor (immune) microenvironment of original human tumors (2). Mice models, which are useful for studying classic drug’s efficacy, cannot be used to evaluate all forms of immunotherapy, because of the considerable differences between the immune system of mice and humans (12). Patient-derived tumor xenografts (PDTX) models can properly recapitulate the interactions of cancer cells with surrounding elements, except the interactions with the immune system. Humanized immuno-oncology models have been generated to overcome this problem. These models bearing human immune cells, but cost, throughput, and complete immunocompatibility, remain challenges (13, 14). On the other hand, the generation of a successful PDTX model is time-consuming, about 4-8 months, therefore, these models cannot be a rational choice for real-time precision cancer therapy (15). The selection of effective transplanting approaches for various tumor tissues and the generation of specific subtypes of tumors are other limitations of PDTXs models (16–19). Given the role of the tumor (immune) microenvironment in the drug screening and immunotherapy studies (20, 21), the presence of pre-existing structural elements such as mouse stromal cells in TME of PDTX models can affect the validity of the study results (20). All the models mentioned above inadequately model the complex immunobiology and pathophysiology of the original parent tumors. In addition, animal models are expensive and time-consuming to develop and apply (22). Hence, it is necessary to develop an alternative model that can recapitulate the human TME while preserving the human immune system components. Therefore, using human tumor organoid models is necessary to tackle these limitations. Indeed, organoid models can properly recapitulate the tumor (immune) microenvironment by preserving tissue architecture, endogenous stromal components including various immune cells, or by adding exogenous immune cells, vasculature, and other components (23–29). Therefore, PDO culture systems could model immunotherapy responses and facilitate the immunotherapy preclinical testing. PDTXs accurately retain the heterogeneity of human tumors, but in contrast to PDTXs, PDOs can be cultured for a long time, expanded, and finally cryopreserved. The establishment of large organoid biobanks has been made possible by the propagation of tumor biopsies *in vitro*, that these tumor biobanks preserve the mutational diversity and histological properties of native human tumors (30–32). Here, we discuss the various organoid culture strategies for the modeling of TIME and the applications of complex PDOs in testing cancer immunotherapeutics and personalized cancer immunotherapy.

## Organoid Culture Techniques for Immunotherapy Studies

Various organoid culture approaches are utilized for modeling the TIME that are suitable for immunotherapy including 1) Reconstitution approaches, such as submerged Matrigel culture, because the typical submerged Matrigel organoids contain exclusively epithelial cells, any study on immunotherapy and TIME in this approach requires the addition of exogenous immune cells and other stromal cell types (2). 2) Holistic approaches, such as air-liquid interface (ALI) and microfluidic 3D culture that in these culture strategies the native TIME and small fragment of tumor tissue, as an intact unit without reconstitution, are preserved (2) (**Table 1**). Methods such as ALI, in which the tissue architecture is preserved, are also known as explant culture methods (48). In addition, 3D micro-sized cell aggregates that are generated as suspension or embedded within a 3D matrix are known as spheroids (49).

**Table 1:** Overview of organoid culture techniques in cancer research.

	Organoid culture techniques		
	Submerged Matrigel culture	Air-liquid interface culture	Microfluidic 3D culture
**Tissue processing before culture**	Tissues are dissociated mechanically or/and enzymatically	Tissue is minced into small fragments	Tissues are dissociated mechanically or/and enzymatically; by filtering 40–100 μm-sized tumor fragments are collected and pelleted in ultra-low-attachment plates
**Culture matrix** **And Culture equipment**	Matrigel Dish or plate	Collagen Inner dish, Outer dish (Transwell plates with permeable membrane inserts)	Collagen Microfluidic device
**Plating condition**	Cells culture underneath medium in mixture with 3D Matrigel	Mixture of tissue fragments and collagen plated on the inner dish with a bottom collagen layer; medium is added into an outer dish that can diffuse into the inner trans-well dish through a permeable membrane; top of collagen layer is exposed to air	Spheroid-collagen mixture is inoculated into central gel region of the device; medium is added into media channels on both sides
**Preserved cell types of original tumor tissue in culture**	Cancer cells exclusively	Cancer cells, tumor-infiltrating myeloid and lymphoid cells, native immune cells, and stromal cells	Cancer cells, tumor-infiltrating myeloid and lymphoid cells, native immune cells, and stromal cells
**Modeling the tumor immune microenvironment**	PBMCs, DCs and other immune cells can be added to the culture	Immune cells of tumor tissue are faithfully preserved	Immune cells can be added in the medium; immune cells of tumor tissue are faithfully preserved
**Advantages**	Organoid expansion is convenient	Cellular complexity and architecture of the tumor tissue are maintained as an intact *en bloc* unit without reconstitution	Cellular diversity and architecture of the tumor tissue are maintained; small amount of medium/reagents and small number of cells are required; it can be automated; mimicking physiological shear flow
**Limitations**	Stromal components and immune cells are usually not preserved in tissue processing stage, determining the growth factors and/or inhibitors required to maintain all subclones is the laborious and time-consuming process; does not reflect the native tumor-infiltrating immune cells, lack of immune components hinders immunotherapy assessment; allogeneic cultures will result in high background killing compared with autologous systems	Determining the growth factors and/or inhibitors required to maintain all subclones is the laborious and time-consuming process; necrosis and hypoxic cores of organoids; the immune components decline over time and do not persist beyond ~2 months	Determining the growth factors and/or inhibitors required to maintain all subclones is the laborious and time-consuming process; size limitation; requires specialized equipment; the immune components decline over time
**Possible future improvements**	Culture duration can be extended; establishing organoid biobanks for model standardization across laboratories; incorporating multiple organoid types into single microchips; using synthetic scaffolds with precise ECM composition that is essential for reproducible research; increase immune cellular complexity by both incorporated into, and preserved in; to overcome to the formation of a necrotic core, and better recapitulation of native TME organoid vascularization and perfusion are needed		
**References**	(33–41)	(42, 43)	(44–47)

### Submerged Matrigel Culture

The submerged Matrigel technique is widely used to culture dissociated cancer cells from tumor biopsies underneath tissue culture medium in a mixture with a dome or flat gel of 3D Matrigel. In this culture method, various growth factors and/or pathway inhibitors are added to the culture medium depending on the type of tumor tissue (25, 50–52). Customized culture conditions have been adapted for many different tissues (25, 32, 53–64), but often include ligands and additives, such as Wnt3a and/or R-spondin, bone morphogenetic (BMP) inhibitor Noggin, and epidermal growth factor (EGF) (25), which allow the stem cells to undergo long-term self-renewal and differentiation into various cell lineages (25). These niche factor requirements are mainly determined by genetic variations that increase tumorigenicity (51). These supplementations are also been utilized in other organoid culture strategies such as ALI (42). Dissociation of tissue during organoid preparation leads to activation of Rho kinase (ROCK)-dependent programmed cell death, therefore the addition of ROCK inhibitors to the medium can efficiently increase the success rates of organoid generation (25, 65, 66). It should be noted that conventional submerged Matrigel methods exclusively enrich epithelial tumor cells, but fail to preserve stromal components (25). Hence, TIME modeling in these techniques needs a co-culture of PDOs with exogenous (immune) cells. Seino et al. (50), by using this technique showed that co-culturing of human pancreatic ductal adenocarcinoma (PDAC) organoids with cancer-associated fibroblasts (CAFs), which shows immunosuppressive activity in TME (67), trigger organoid growth of WNT-nonproducing PDAC subtypes by CAF produced WNT (50). On the other hand, TGFβ and IL-1 ligands secreted by PDAC organoids can increase CAF heterogeneity and induce distinct myofibroblast and inflammatory CAF subtypes, respectively (68). Therefore, selective targeting of tumor-promoting CAFs can be improved by understanding the CAF heterogeneity mechanisms (68). Chakrabarti et al, designed a complex submerged Matrigel culture system by co-culturing of mouse tumor organoids with cytotoxic T lymphocytes and bone marrow-derived dendritic cells pulsed by conditioned media (tumor antigen) collected from tumor organoids. The promotion of apoptosis by activated CTLs was observed in cancer cell in the presence of PD-L1 neutralizing antibody (69). This method, co-culture of immune cells and human tumor organoids, is also used for the generation of tumor-reactive T cells from autologous peripheral blood lymphocytes (33) and survey on the *Helicobacter pylori* infection process (34, 70). Forsythe et al. (71), utilized a collagen-based extracellular matrix (ECM, hydrogel) to fabricate PDOs. They generate immunocompetent organoids with coculturing of tumor cells with patient-matched immune cells derived from peripheral blood mononuclear cells (PBMC), spleen, and lymph nodes. They showed that immunocompetent organoids can be useful in the preclinical study of personalized immunotherapy efficacy.

### Microfluidic 3D Culture

In microfluidic 3D devices, murine- or patient-derived organotypic tumor spheroids (MDOTS/PDOTS) are cultured in a mixture of collagen gel (72). For MDOTS/PDOTS culture (44), tumor tissues specimen is obtained from the patient and dissociated mechanically and enzymatically. This procedure ultimately gives a heterogeneous mixture of single cells, spheroids, and macroscopic tumor fragments. Then, this mixture is filtered through 100 μm and 40 μm filters to obtain spheroids with 40–100 μm in diameter, afterward, this fraction is pelleted in ultra-low-attachment plates and mixed with collagen gel, and inoculated into the center region of the microfluidic device. To feed the spheroids, the culture medium is added into the media channels located on both sides of the central channel. In this approach, spheroids maintain the native cancer tissue complexity and cellular diversity such as autologous myeloid populations (tumor-associated macrophages [TAM], monocyte, DC, and MDSC), lymphocytes (B cell and T cell), and cancer cells without reconstitution (45). T cell infiltration into cancer spheroids and tumor-immune cell interactions and cross-talk can be studied in these devices by adding exogenous T cells such as Jurkat cells into the media channels (46, 73). The composition of the devices and the size of media and central channels are variables that can reduce the validity and reproducibility of studies (44). The composition of the devices such as PDMS (polydimethylsiloxane) is one of the interfering factors in testing immune checkpoint blockade (ICB) in a mixture with small molecules (generally prepared in dimethyl sulfoxide, DMSO), because PDMS can adsorb the small hydrophobic molecules (74) that prevent the drug delivery to tumor spheroids.

### Air–Liquid Interface Culture

In this approach, two inner and outer dishes are used. The inner dish consists of two layers, bottom and top, for preparation of the bottom layer the collagen gel matrix was added to the inner dish (43). For the preparation of primary tissues, after obtaining the tissue specimen, it immediately are placed in an ice-cold medium (43). After rinsing the tissue, it mechanically dissociated into small fragments and then are cultured in a mixture with collagen gel as a top layer of the inner transwell dish by pouring the mixture onto the inner dish with the bottom layer gel matrix (43). Then prepared inner dish is placed into an outer dish, transferred to a 37°C incubator, and allowed the gel of the inner dish to solidify (43). Afterward, culture media is added to the outer dish that can diffuse into the inner dish through a permeable membrane. In addition, the top layer of culture is in direct air exposure that supply tissue organoid oxygen efficiently (26, 43, 75). ALI allows the growth of large multicellular tissue fragments that retain native tissue architecture, such as cancer cells *en bloc* with endogenous stromal and immune cells without reconstitution, which is different from submerged Matrigel culture (76). In addition, ALI PDOs preserve not only the architecture and complex cellular composition of the TME, but also the genetic alterations of the native tumor (42). These features of ALI, in contrast to submerged Matrigel Culture, make it suitable for TME modeling (32). It has been observed that in ALI, probably essential endogenous niche factors produced by stromal cells are sufficient to support tissue fragment growth without growth factor supplementation (26).

### Organoid On-a-Chip

Organoid culture without physical restrictions resulted in organoid fragments with differences in shape, size, geometry, and cell number. As well, the mechanical factors such as fluid shear stress, tension, and compression are not recapitulated the native condition of tumor in 2D or 3D culture models. Therefore, these limitations lead to the non-reproducibility of results in organoid cultures (3). Organoid-on-a-chip models can overcome this challenge by increasing of uniformity of organoids and mimicking the physical conditions of the body such as providing perfusion of culture media. In addition, microfluidic devices can monitor and control the culture condition and assay variables by integrated sensors and actuators (77). The multi-organoid-on-a-chip have also been built (77, 78), nevertheless, optimization of culture media and mechanical condition for different tissue remain as a major challenge.

## Complex Organoids for Immunotherapy

For various reasons, the TME is not fully recapitulated in most organoid culture methods. This drawback, especially the lack of immune cells, limits the application of organoids in immunotherapy studies. Nevertheless, many studies are underway to solve this issue.

### Adding Immune Components

In some cases, immune cells are not added, but the immune cells within the organoid are maintained and expanded. Zumwalde et al. (79), showed the presence of immune cell populations and characterized the intraepithelial lymphocyte compartment within the organoid culture. They demonstrated that leukocyte populations of the breast organoid differed from those in peripheral blood, and conserved T lymphocytes can be expanded in response to the bisphosphonate. As noted above, cultured tumor spheroids in microfluidics devices are able to preserve autologous myeloid and lymphoid cell populations (45). The air-liquid interphase (ALI) culture strategy is another approach that retains native immune and stromal components. In ALI, the cancer epithelium and its complex microenvironment including fibroblasts, immune cells such as cytotoxic T lymphocytes (CTLs), helper T cells, TAMs, natural killer (NK) cells, B cells, and NK T cells are maintained for over a 1- to 2-month period (42). In addition, the T cell receptor (TCR) spectrum of the native tumor is accurately preserved in organoid culture (42).

In some organoid culture strategies, such as the submerged Matrigel technique, stromal and immune cells are not preserved. Therefore, to perform immunotherapy studies, it is necessary to add immune cells to the organoid. Tsai et al. (80), generated pancreatic cancer organoids using primary organoid co-culture with patient-matched peripheral blood lymphocytes and CAFs that were relevant to the immunotherapy and tumor-immune cell interaction studies. Activation of myofibroblast-like CAFs and tumor-dependent lymphocyte infiltration were detected in these complex organotypic models. In another study (33), co-cultures of autologous tumor organoids and peripheral blood lymphocytes were established for the enrichment of tumor-reactive T cells from peripheral blood of colorectal cancer (CRC) and non-small-cell lung carcinoma (NSCLC) patients. Thus, this approach can be used to evaluate the efficiency of the killing of tumor organoids by autologous tumor-reactive T cells (33). Many factors can affect the outcome and our success in the generation of complex organoids. For instance, in some cancer immune cells located in the surrounding stroma of the tumor or completely lack in TME. Therefore, this will impact the immune cell population of primary organoid culture (3). Furthermore, the addition of exogenous immune components to organoids will not be able to recapitulate the complexity of the patient-specific immune cell composition within TME. If allogeneic immune cells are used as a source of exogenous immune components, because of interpersonal differences in HLA, it will lead to high background killing compared with patient-specific PBMCs (3). Ultimately, choosing the proper strategy for co-culture depend on the downstream assays.

### Organoid Vascularization and Perfusion

Lack of perfusion flow and vascular networks in organoids remains a major challenge in the preparation of complex organoids as an ideal cancer model. The absence of vasculature limits the size of the organoid, and organoids larger than 100-200 μm in diameter suffer from the diffusion of nutrients, oxygen, and metabolites to the central region of the organoid. Furthermore, organoid fragments larger than 500 μm in diameter show necrosis (81, 82). Many studies have been performed to overcome this problem and establish perfusion flow in the organoid (23, 27–29). Wörsdörfer et al. (29), co-cultured the mesodermal progenitor cells (MPCs) with tissue-specific (progenitor) cell types for the establishment of complex neural as well as human tumor organoids. They showed the directed incorporation of MPCs into organoids and the formation of vascular networks in these organoids. They observed the expansion of vascular networks during organoid growth. In addition, this endothelial network was responsive to pro-angiogenic conditions and anti-angiogenic agents (29). Organoid vascularization has been previously well-reviewed by Grebenyuk and Ranga (28).

## Application of Organoids in Immunotherapy

**Figure 1:**
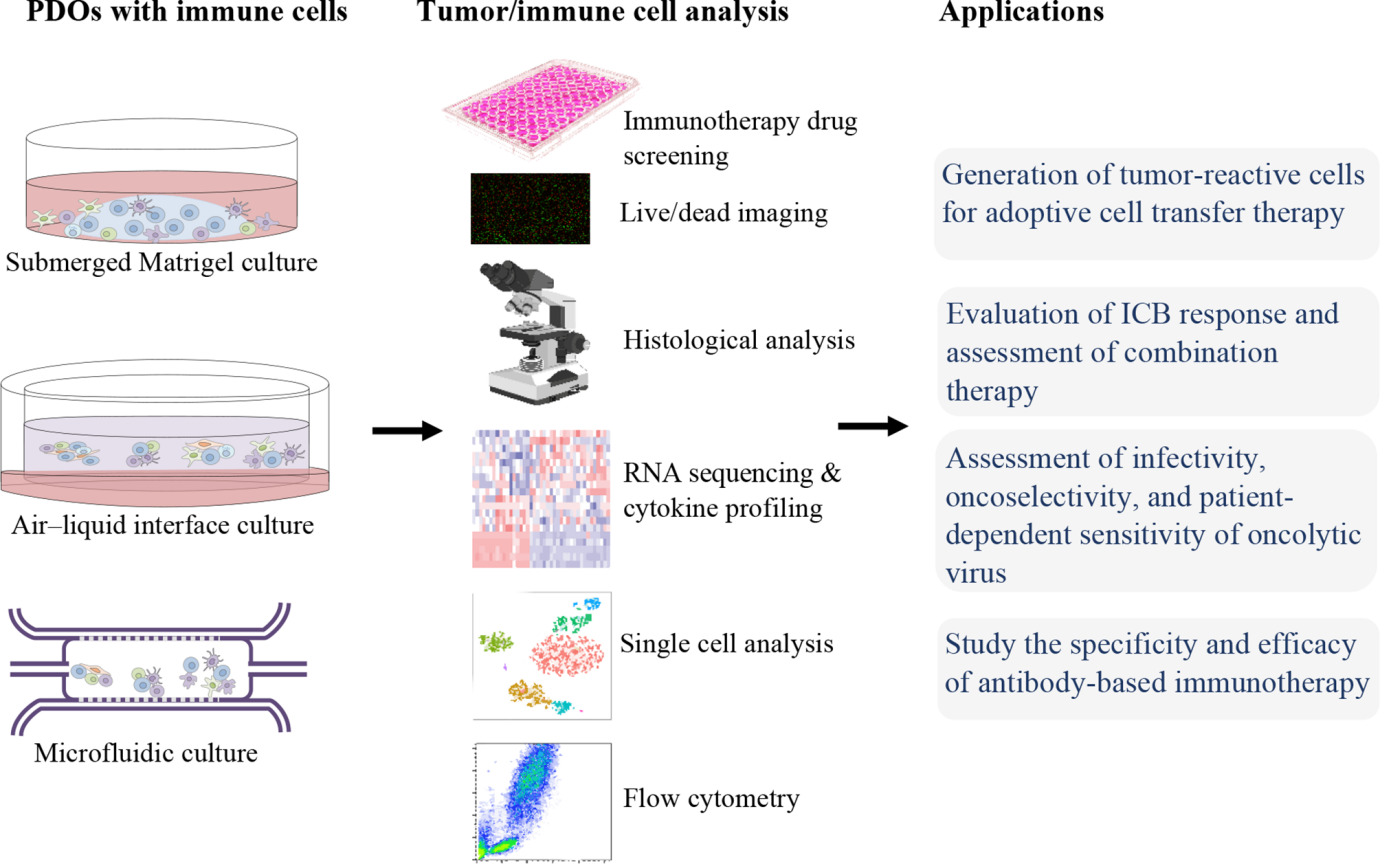
Patient-derived organoids (PDOs) for precision cancer immunotherapy.

For immunotherapies screening and studies, an ideal *ex vivo* model is needed to be able to fully recapitulate the heterogenicity of the native TME. For this reason, PDOs, which can preserve TIME as well as represent the stage and the treatment history of the patient, can be utilized. In the following, we will discuss the applications of organoids in immunotherapy (**Figure 1**).

### Adoptive Cell Transfer Therapy

In ACT immunotherapy, TILs or circulating lymphocytes are collected, high-affinity TCRs recognizing tumor antigens are selected or genetically engineered, subsequently, these cells expanded and activated *ex vivo* followed by reinfusion into patients (8, 83). CAR T cells are genetically modified T cells that produce an artificial T-cell receptor, targeting a specific antigen on the surface of tumor cells and circumventing MHC restriction (84). Studies have shown that PDOs are efficient platforms to evaluate the tumor-specific cytotoxicity of T cells (e.g., CAR T cell, TCR T cell). In the study of Michie et al. (85), PDOs were exploited to evaluate the effect of combination therapy of CAR T cells and birinapant, the inhibitor of apoptosis antagonist. They observed that the combination of CAR T cells with birinapant significantly reduce PDOs growth in a tumor necrosis factor (TNF)-dependent manner, while CAR T cells alone were relatively ineffective. Schnalzger et al. (35), developed a sensitive preclinical model, 3D PDOs, that allows assaying the CAR-mediated cytotoxicity in the native TIME mimicking model. Furthermore, they established a confocal live-cell imaging protocol for dynamic monitoring of cytotoxic activity toward organoids at a single organoid level. They demonstrated a stable effector-target cell interaction in the co-culture of NK cells with CRC or normal organoids on an ECM layer. In addition, CRC organoids were utilized to monitor the tumor antigen-specific cytotoxicity of EGFRvIII or FRIZZLED receptors-targeting CAR-engineered NK-92 cells. In sum, they established a sensitive platform to assay CAR efficacy and tumor specificity in a personalized manner (35). In addition, Epithelial-only PDOs, while lacking stromal and immune components, can be used for the selection of tumor-reactive T cells (33). This co-culture strategy can be served to enrichment, stimulation, and efficacy evaluation of tumor-reactive lymphocytes. Dijkstra et al. (33), cocultured CRC or NSCLC organoids with autologous circulating T cells (PBMCs), in medium supplemented with anti-PD1, anti-CD28, and IL-2 for generation of tumor-reactive CD8^+^ populations. After two weeks of co-culture, T cell-mediated killing, MHC-dependent cytotoxicity, against autologous tumors organoids, and upregulation of CD107a and IFNγ secretion in CD8^+^ T cells were observed. But, these tumor-reactive CD8^+^ cells did not affect the survival of matched healthy organoids. Therefore, they established a platform for the expansion of tumor-reactive T cells and evaluation of the specificity and sensitivity of cancer cell killing by autologous T cells in a personalized manner. Additionally, tumor-reactive T cells can be isolated from TILs that are more specific than the non-infiltrating lymphocytes, subsequently, these cells can be infused back into the patient (86).

### Immune Checkpoint Inhibitors

Clinical benefits of ICIs that target CTLA-4, PD1/PD-L1 have been observed in various advanced tumors, such as renal cell carcinoma (RCC) (87), cutaneous squamous cell cancer (88), melanoma (11), head and neck cancer (89), and NSCLC (10). Epithelial-only organoid biobanks have been established from diverse malignancies and are widely available through entities such as the Human Cancer Models Initiative (HCMI). But lack of immune compartments in these types of PDOs hinders their immunotherapy application. Some studies have been performed to overcome this issue. For instance, Kong and colleagues (36), cocultured epithelial-only submerged Matrigel organoids with autologous TILs and demonstrated the TILs migration toward organoids and cytotoxic activity of T cells. They also showed the rescue of TIL function after ICB. But since, co-culturing the epithelial only PDOs with exogenous immune cells, as a reconstitution approach, cannot fully recapitulate the complex interaction and crosstalk between diverse cell populations in the TME, especially when using immunomodulatory drugs. To tackle this problem, a holistic culture approach, such as 3D microfluidic and ALI culture strategies, can be utilized for TIME modeling. The dynamic response and resistance to ICB (such as PD-1 blockade) can be recapitulated using organotypic spheroids in a short-term 3D microfluidic culture that maintains autologous myeloid and lymphoid cell populations similar to the original donor human or mouse tumors (45). As well, the cytokine secretion profiles of PDOTS/MDOTS matched the profile of the donor tumors (45). Therefore, PDOTS/MDOTS profiling facilitates the evaluation of ICB using clinically relevant models. Neal et al. (42), utilized WENR (WNT3A, EGF, NOGGIN, and RSPO1) base medium to expand and serially passage physically processed cancer fragments as ALI organoids. They demonstrated that ALI PDOs, like the microfluidic approach, preserve the stromal and immune cell populations, and effectively recapitulate the expansion, activation, and tumor cytotoxicity of tumor antigen-specific TILs in response to PD-1/PD-L1 ICB. They observed the CD8^+^ TIL expansion, activation, and tumor cell killing after 1 week of anti-PD-1 treatment of ALI PDOs from various human tumor biopsies, including RCC, NSCLC, and melanoma. It should also be noted that the material and composition of devices used in organoid culture can affect the results of immunotherapy such as ICB studies (74).

### Other Immunotherapy Applications

PDOs can be used as predictive tools to study the specific infectivity, and cytotoxicity of the oncolytic virus alone or in combination with chemotherapy (90). In the study of Raimondi et al, Oncolytic adenovirus (OA) displayed a good selectivity of replication in PDAC organoids, but not in healthy pancreas tissue organoids. Patient-specific responses were also observed, indicating that PDOs are reliable *in vitro* tumor models to assay preclinical responses to oncolytic viruses.

Tumor organoids can also be utilized to study the specificity and efficacy of antibody-based immunotherapy. As mentioned in the previous section, many studies used the organoid models in antibody-based ICB therapies (42, 45, 69, 91). Courau et al. (92), showed that both NKG2D-MICA/B and NKG2A-HLA-E pathways are involved in the infiltration process of activated/memory T and NK cells into organoids, subsequently these activated cells can kill cancer cells and disrupt the 3D structure. They demonstrated that anti-MICA/B and a combination of both anti-MICA/B and anti-NKG2A antibodies were able to induce immune-mediated destruction of colorectal tumor organoids during cocultures with autologous TILs. Gonzalez-Exposito et al. (93), established seven PDOs from treatment-refractory metastatic CRC and one from a treatment naïve primary CRC, to investigate on resistance and sensitivity mechanisms of cibisatamab, a bispecific monoclonal antibody that binds carcino-embryonic antigen (CEA) on tumor cells and CD3 on T cells. They designed a co-culture of organoids and allogeneic CD8^+^ T cells to evaluate cibisatamab efficacy. Using this platform, they demonstrated that CEA_low_ PDOs were resistant whereas CEA_high_ PDOs were sensitive to cibisatamab. Indeed, CEA_low_ cells maintain tumor cell growth, and an increased WNT/β-catenin pathway activity was detected in CEA_low_ cells by RNA-sequencing. They suggested the use of WNT/β-catenin pathway inhibitors in combination with cibisatamab as a potential strategy to increase the treatment success.

## Conclusions

Nowadays, the use of PDOs to study the dynamic interactions between cancer and the immune system has attracted increasing attention. In addition, the modeling of TME could facilitate immunotherapy screening in the preclinical setting. Lack of stromal components and vascular network are the major limitations of organoid technology for studying the influence of TIME on cancer behavior against immunotherapy agents. To overcome these restrictions, the complex organoids have been developed by co-culturing of organoids (or progenitor cells) with immune cells, CAFs, mesodermal progenitor cells. In addition, co-culturing of organoids with PBMCs or immune cells from lymph nodes can model the cancer-immunity cycles, including effector T cell priming/activation, T cell trafficking/infiltration into tumor tissues, and recognition/killing of cancer cells by T cells. For the long-term preservation of immune cells, using additional supplements such as anti-CD28, anti-CD3, and IL-2 antibodies has been suggested. As well, the culture media composition should be optimized in such a way that supports the growth of all clones without selective growth of specific colonies. Recapitulation of mechanical stress such as physiologic shear flow can improve the modeling of native TME. Using scaffolds with well-defined composition and control of size, shape, cell number, and relative arrangement of different cell types within organoids could improve the reproducibility of drug screening results. In comparison to animal models, establishing a research platform with organoid models take less time: it is feasible to establish a successful human organoid culture within a few weeks or months, as a result, it is possible for PDOs to be used for precision medicine to provide robust data on individual mutation profiles and drug responses (94). Numerous clinical trials are underway to evaluate the various applications of organoids and their effectiveness in precision cancer immunotherapy.

## Author Contributions

C-PS: Conceptualization, Writing-original draft, Writing-review and editing. H-RL: Validation, Writing-original draft, Writing-review and editing. X-LF: Funding acquisition, Validation, Writing-review and editing. X-YY: Supervision, Validation, Writing-review and editing. K-TJ: Funding acquisition, Supervision, Writing-review and editing. All authors contributed to the article and approved the submitted version.

## Funding

This work was supported by National Natural Science Foundation of China (grant no. 82104445 to H-RL), Zhejiang Provincial Public Welfare Technology Application Research Project (grants no. LGF22H160046 to H-RL, and LGF21H160004 to X-LF), Jinhua Municipal Science and Technology Projects (grants no. 2021-3-040 to K-TJ, and 2021-3-046 to H-RL).

## Conflict of Interest

The authors declare that the research was conducted in the absence of any commercial or financial relationships that could be construed as a potential conflict of interest.

## Publisher’s Note

All claims expressed in this article are solely those of the authors and do not necessarily represent those of their affiliated organizations, or those of the publisher, the editors and the reviewers. Any product that may be evaluated in this article, or claim that may be made by its manufacturer, is not guaranteed or endorsed by the publisher.
